# HEAVY ALCOHOL CONSUMPTION IN MIDLIFE IS ASSOCIATED WITH ACCELERATED BRAIN AGING SIX YEARS LATER

**DOI:** 10.1093/geroni/igz038.3324

**Published:** 2019-11-08

**Authors:** Riki E Slayday, Carol E Franz, Sean N Hatton, Linda K McEvoy, Michael J Lyons, William S Kremen

**Affiliations:** 1 San Diego State University, San Diego, California, United States; 2 University of California, San Diego, San Diego, California, United States; 3 Boston University, Boston, Massachusetts, United States

## Abstract

Excessive alcohol consumption is associated with cognitive decline, exacerbated brain atrophy, and dementia in older adults, but associations with midlife brain health are less well understood. We hypothesized that heavy drinkers would have older-looking brains in late midlife. We examined alcohol consumption at mean age 56 (range 51-59) in 364 men from the Vietnam Era Twin Study of Aging (VETSA) and their predicted brain age at mean age 62 (range 56-67). We created five midlife alcohol consumption groups based on drinks consumed over the past two weeks: never, former, light (1-14), moderate (15-28), and heavy (>28). Participants underwent structural magnetic resonance imaging at mean age 62. Predicted brain age was measured using the Brain-Age Regression Analysis and Computation Utility software (BARACUS). Models adjusted for age, scanner, race/ethnicity, SES, smoking, health, depressive symptoms, alcohol dependence, general cognitive ability at age 20, and non-independence of twins within pairs. Heavy drinkers had a significantly older predicted than chronological brain age (M= 5.93, SE= 0.88) compared to each of the other four groups (p’s < 0.05). There were no significant differences among the never (M= 2.88, SE= 0.98), former (M= 2.76, SE= 0.74), light (M= 3.00, SE= 0.94), or moderate (M= 5.93, SE= 0.88) consumption groups. Heavy alcohol consumption at age 56 was associated with an approximately 3-year greater predicted brain age difference at age 62. There was no evidence of protective effects of light/moderate drinking over non-drinking. The neurotoxic effects of excessive alcohol may exacerbate brain aging in late midlife.

